# Using computer-aided design/computer-aided manufacturing technology for foreign body removal from soft tissues: a case report

**DOI:** 10.1186/s40902-025-00479-4

**Published:** 2025-09-30

**Authors:** Teruhide Hoshino, Shuji Yoshida, Chihiro Kurihara, Koki Oiwa, Kotaro Tachizawa, Keisuke Sugahara, Akira Katakura

**Affiliations:** 1 Department of Dentistry and Oral Surgery, National Hospital Organization Tokyo Medical Center, Tokyo, Japan; 2 Department of Oral Pathobiological Science and Surgery, Tokyo dental College, Tokyo, Japan; 3 Department of Oral and Maxillofacial Surgery,, Tokyo Dental College, Tokyo, Japan

**Keywords:** Computer-assisted surgery, 3D-printed guide, Buccal soft-tissue foreign body

## Abstract

**Background:**

When foreign bodies are located deep within the tissue, removal is often difficult. In recent years, computer-assisted surgery (CAS) has been widely adopted in surgery, including the removal of foreign bodies. Among various techniques, computer-aided design/computer-aided manufacturing (CAD/CAM) technology has been widely employed for hard tissue management in the oral and maxillofacial region, and reports on the application of CAD/CAM technology for procedures involving soft tissues are lacking. In this study, we report a case in which CAD/CAM technology is used to facilitate the removal of a foreign body located in the soft tissue.

**Case presentation:**

A 25-year-old female underwent metal plate and screw removal in 2020; however, a metal fragment remained lodged in the buccal soft tissue on the left side of the lower jaw. Computed tomography (CT) revealed a high-density area, indicating a metal fragment measuring 1.5 × 1.3 × 2.5 mm. We identified the exact location of the fragment using preoperative CT data processed with Mimics^®^ (Materialise). A surgical guide was designed using Magics^®^ (Materialise) and fabricated using a 3D printer, enabling precise identification of the vertical and horizontal positions of the foreign body within the soft tissue. The use of CAD/CAM technology facilitated accurate localization and rapid removal of the fragment. The operative time of removal was 1 h and 5 min. Minimal bleeding occurred, and the postoperative course was uneventful, with no signs of infection or nerve damage.

**Conclusions:**

This case demonstrates the successful application of CAD/CAM technology for the identification and removal of a foreign body from soft tissue.

**Supplementary Information:**

The online version contains supplementary material available at 10.1186/s40902-025-00479-4.

## Background

In the area of oral and maxillofacial surgery, cases of foreign bodies migrating into tissues are occasionally encountered [1, 2]. When such foreign bodies are located deep within the tissue, removal is often difficult. In recent years, computer-assisted surgery (CAS) has been widely adopted in surgery. Among various CAS methods, computer-aided design/computer-aided manufacturing (CAD/CAM) technology has been widely employed for hard tissue management in the oral and maxillofacial region [3–5]. CAS has been used to remove foreign bodies from hard tissues; however, reports on its application in soft tissue procedures are lacking. There are no reports specifically regarding the removal of foreign bodies located in the tissue around the cheek. Therefore, we present a case in which CAD/CAM technology was utilized to facilitate the removal of a foreign body that dislodged and became embedded in the soft tissues during orthognathic surgery.

## Case presentation

**Fig. 1:**
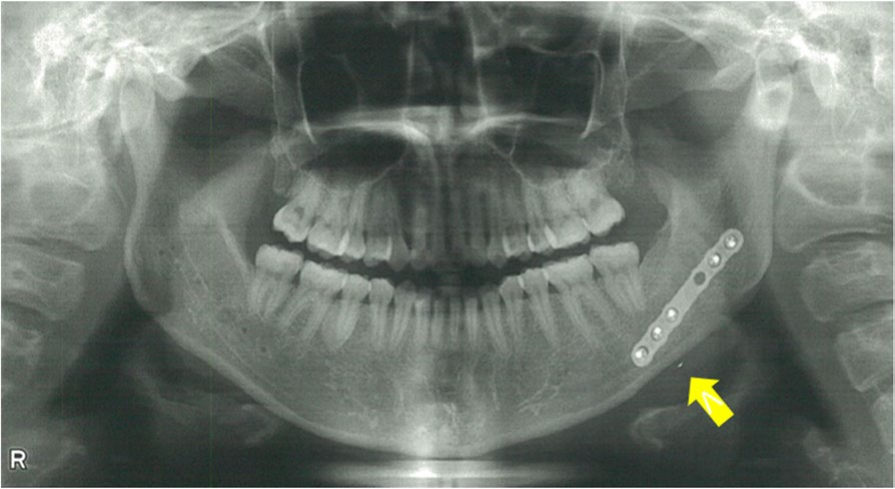
Panoramic radiograph after the first metal plate removal surgery (arrow: metal fragment)

A healthy 25-year-old female presented with a chief complaint of discomfort in the left cheek region. She had previously undergone bilateral mandibular sagittal split osteotomy in 2015. In 2020, she underwent an initial oral surgery at another institution to remove the metal material following satisfactory bone healing. However, a metal plate on the left side could not be removed, and a metal fragment remained embedded in the buccal soft tissue of the left side of the lower jaw (Fig. 1). Therefore, the patient underwent a second surgery to remove the metal plate on the left side in 2020; however, this surgery was unsuccessful. Subsequently, in February 2021, the patient visited a general dental clinic specializing in oral and maxillofacial surgery, where a third surgical attempt was made to remove the metal fragment. However, the procedure was interrupted owing to difficulties in locating the fragment. In May 2021, the patient presented to the Department of Oral and Maxillofacial Surgery at Suidobashi Hospital, Tokyo Dental College, Japan.

**Fig. 2:**
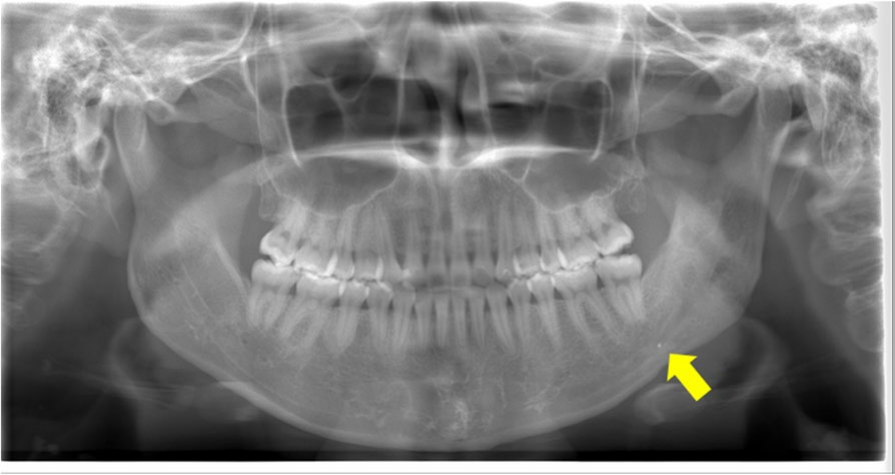
Panoramic radiograph at the first visit. A radiopaque object can be observed near the apices of the mandibular left molars (arrow: metal fragment)

**Fig. 3:**
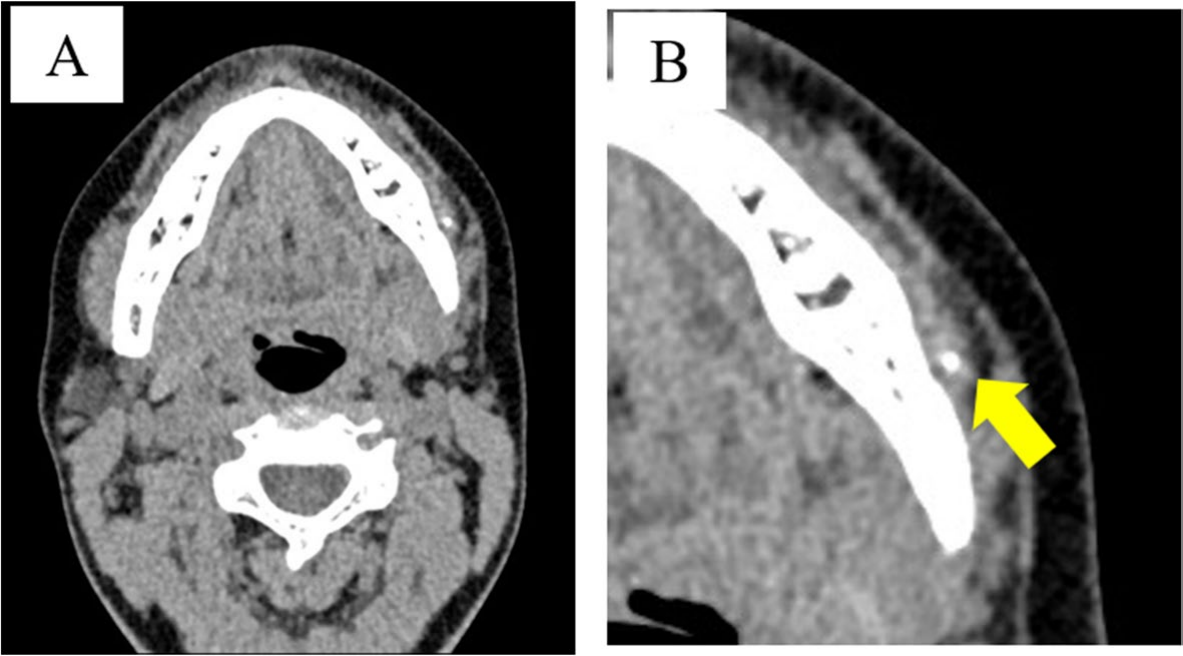
Computed tomography image (horizontal section). **A** A high-concentration area suggestive of a metal fragment measuring approximately 1.5 × 1.3 × 2.5 mm can be observed near the posterior margin of the inferior angular control muscle at the lateral osteotomy. **B** Enlarged view (arrow: metal fragment)

**Fig. 4:**
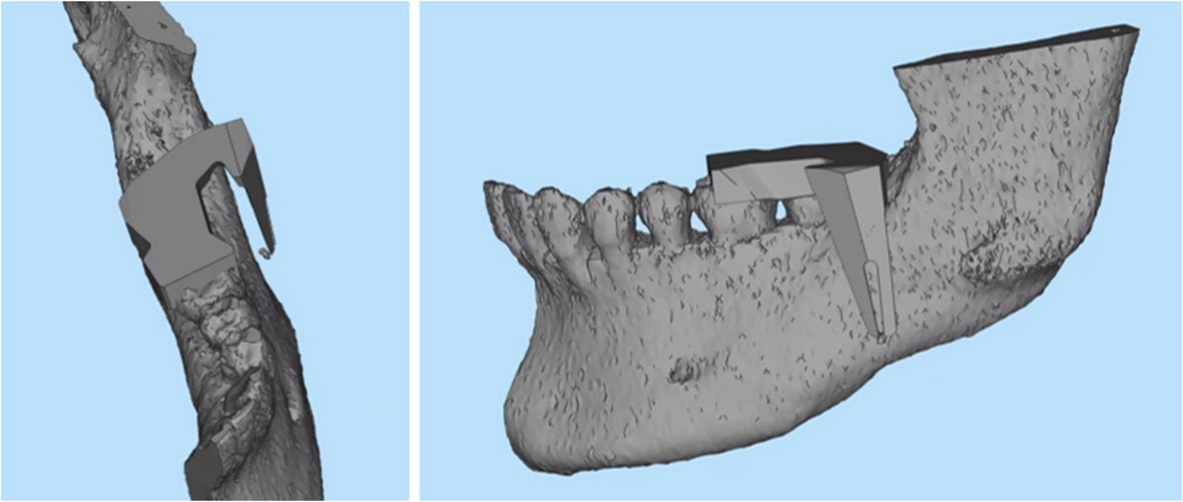
Blueprints using CAD using Magics^®^ (Materialize)

**Fig. 5:**
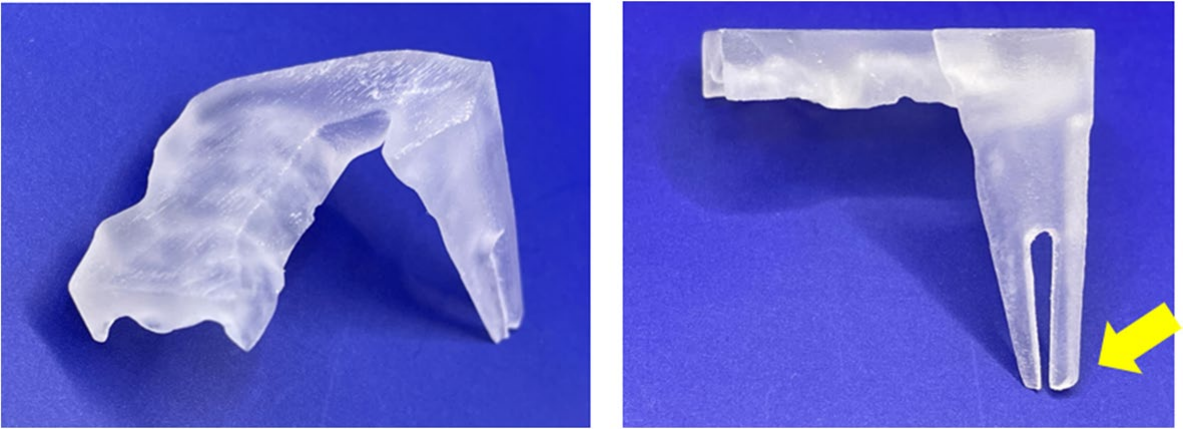
CAD/CAM technology used to fabricate a surgical guide. The surgical guide was fabricated using a 3D printer (arrow: designed to secure the view of the surgical field and mark the position of the foreign body). CAD/CAM, computer-aided design/computer-aided manufacturing

**Fig. 6:**
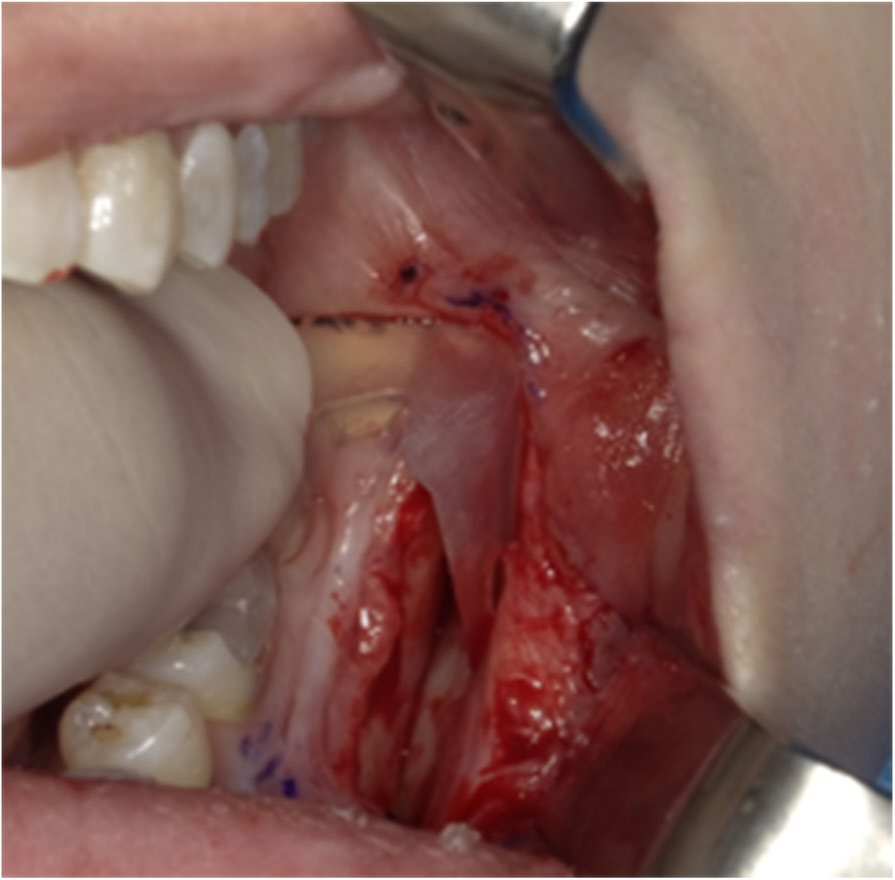
Surgical guide seated in the oral cavity

**Fig. 7:**
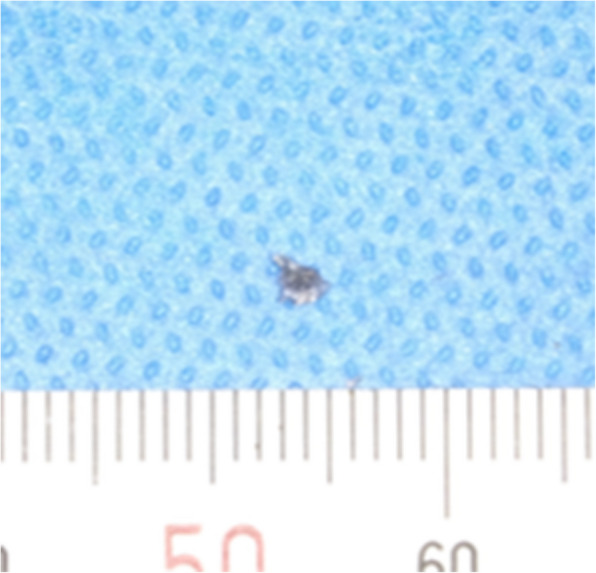
The metal foreign body

**Fig. 8:**
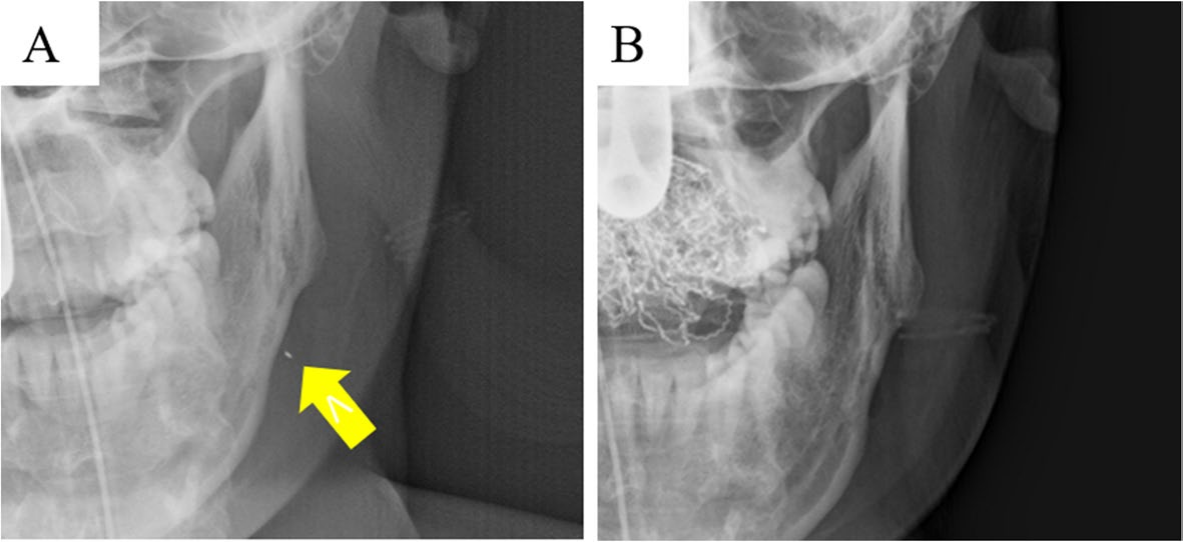
Computed tomography images taken 1-year post-surgery (**A** axial plane, **B** coronal plane). **A** Preoperative (arrow: metal fragment) and **B** postoperative radiographs

**Fig. 9:**
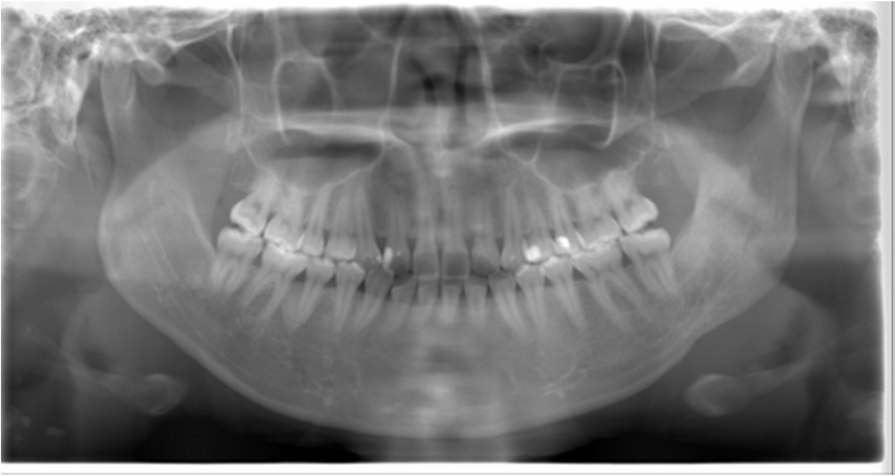
Panoramic radiograph taken the next day after the excision. No radiopaque objects are visible

Panoramic radiography revealed a radiopaque object on the left side of the lower jaw, adjacent to the root apices of the molars (Fig. 2). Computed tomography (CT) revealed a high-density area, suggesting the presence of a metal fragment measuring approximately 1.5 × 1.3 × 2.5 mm near the posterior margin of the depressor anguli oris muscle (Fig. 3A, B). The imaging conditions were set with a slice width of 0.6 mm and a slice pitch of 0.8 mm. In this case, the patient had no dental crown restorations, and no artifacts from restorations were observed. Furthermore, we addressed the situation by guiding the patient’s head into the appropriate position, and she was instructed not to move. In December 2021, the patient reported discomfort in the left cheek region; therefore, the displaced foreign body was removed from the soft tissue under general anesthesia. Hypotensive anesthesia was maintained to minimize bleeding. The surgical guide was prepared in the same manner as in previous cases reported by our department using CAD/CAM technology [3]. The position of the foreign body was confirmed using preoperative CT data, which were processed using Mimics^®^ (Materialise). Following segmentation, a surgical guide was designed using Magics^®^ (Materialise) (Fig. 4). These data were converted into STL format, and the surgical guide was fabricated using a 3D printer (Objet260 Connex; Stratasys Ltd., Eden Prairie, MN, USA). The side tip of the surgical guide was shaped to secure the field of view for the surgeon and mark the position of the foreign body (Fig. 5). The guide was set on the left mandibular molar. The surgical guide was disinfected with povidone-iodine before the surgery. The surgical guide was accurately seated during the operation (Fig. 6). The periosteum, scar tissue, and muscle were excised anteroposteriorly at the same vertical level as the foreign body identified on imaging. Centrifugal traction was applied to expose the wound and secure the surgical field. We searched for the foreign body in the excised tissue and promptly identified it in the anterior muscle layer (Fig. 7). No metal fragments were found on radiographs in the operation room (Fig. 8A, B) and the next day (Fig. 9). “FabLab TDC” is the first digital fabrication laboratory for dental purposes in Japan, established in 2013 [6], and has supported the development of various 3D models and devices, which have been previously reported [3, 7–10]. The operative time of removal was 1 h and 5 min. Minimal bleeding occurred (Table 1), and the postoperative course was uneventful, with no signs of infection or nerve damage. The surgical wounds healed without complications.

**Table 1:** Item and operative metrics

Item	Operative metrics
Estimated blood loss	A little
ASA-PS	I
Operative time	1 h and 5 min
Amounts of blood loss	A little
Postoperative periods of follow-up	2 months

Complications of orthognathic surgery may include displacement of surgical instruments [11], infection, and bone resorption associated with silicone implants in the mandible [12, 13]. Metal fragment removal is associated with a risk of residual metal fragments as the surrounding bone often gets scraped. In this case, a metal fragment was displaced into the soft tissue during metal plate removal. Foreign bodies left in situ pose a potential risk of infection and, if sharp, can cause direct tissue damage depending on the site. Prior to presentation at our institution, the patient had undergone repeated examinations and surgical attempts at three different facilities and experienced severe discomfort in the left cheek. She also expressed considerable anxiety regarding the risk of infection associated with the retained foreign body. Considering both her physical symptoms and mental state, we opted to proceed with surgical removal.

The size and region of displacement are crucial factors when removing foreign bodies; therefore, accurate localization using imaging is essential. In recent years, navigation systems have been employed to assist in foreign body removal from relatively low-mobility regions such as the maxilla, temporal region, and orbits [1]. Similarly, 3D simulations for removal of foreign bodies in the mandible have been reported [2]. However, reports on using CAS for foreign body removal from mobile soft tissues, such as the cheek, have been limited. Soft tissue mobility can complicate the intraoperative localization of foreign bodies, potentially delaying detection. In such cases, CAS may offer a valuable solution. However, navigation accuracy is affected by the patient’s head position, requiring time-consuming intraoperative registration, calibration, and skilled operators. In recent years, splints have been fabricated in advance as a countermeasure against fluctuating head positions, and reference points have been employed to improve registration accuracy [1].

Therefore, we decided to fabricate a surgical guide with the molars as reference points. The fabricated surgical guide allowed the identification of the vertical and horizontal positions of the foreign body in the soft tissue. The side tip of the surgical guide was shaped and designed to mark the position of the foreign object and to preserve the visibility of the surgical field, thereby facilitating quick identification and removal of the foreign body. Although intraoral scans can significantly enhance the accuracy of matching STL data extracted from CT scans, we were unable to perform intraoral scanning in this case due to time constraints and limited patient visits. Furthermore, considering the absence of metal crown restorations in the mouth, we determined that the CT data alone were sufficient to fabricate a reliable surgical guide.

Challenges during foreign body removal include the deviation from the actual size upon CT imaging owing to the influence of metal artifacts, as well as difficulties in securing a clear surgical field owing to bleeding. To address these issues, it is essential to secure a wide surgical field and minimize intraoperative bleeding. In the current case, the periosteum and mucosa were inverted widely and carefully to suppress bleeding. In addition, three attempts to remove the foreign body at other hospitals had resulted in the formation of scar tissue, necessitating meticulous dissection. To further optimize surgical conditions, we collaborated with a dental anesthesiologist to maintain low blood pressure during general anesthesia and prevent intraoperative hemorrhage.

These combined measures allowed foreign body removal with minimal invasiveness and a short surgical time. Using the same imaging method consistently was important, not only for comparative confirmation after foreign body removal but also for determining the position intraoperatively if the foreign body was not detected within the tissue.

Based on the findings of this case report, we propose that CAD/CAM technology, already established in hard tissue surgery, may also be an effective tool for localizing and removing foreign bodies embedded in soft tissue. The use of teeth as markers allowed for a reproducible reflection of the information from the preoperative CT scan in the surgical field. However, the use of a surgical guide may introduce errors regarding the precise location of the foreign body by expanding the surgical field. Recently, changes in soft tissues have been simulated before and after surgery [14]. Fabricating an appropriate surgical guide that reflects the soft tissue dynamics pre- and postoperatively can improve accuracy. In our case, we believe that marking the vertical and horizontal positions using the surgical guide facilitated the successful removal of the metal fragments.

To strengthen the usefulness of surgical guides for the removal of foreign bodies in the soft tissue, additional clinical cases need to be studied. Given that the number of such cases at a single facility is limited, future studies should involve multiple facilities.

The metal fragments observed preoperatively were identified within the excision along with the surrounding tissue, and no obvious metal fragments were identified on postoperative panoramic X-rays. Therefore, we considered the metal fragments to have been completely removed; thus, no postoperative CT scan was performed. However, to more clearly demonstrate successful removal, it may be necessary to add quantitative image analysis using CT scans. We would Like to include this in similar cases in the future. Additionally, we were only able to follow up for 2 months after the surgery since the patient has changed workplaces. However, we believe it would be beneficial to set a slightly longer follow-up period to evaluate the condition of the surgical site and the degree of discomfort.

In this case, we only used CAD/CAM technology; however, we also believe that combining CAD/CAM and MR technologies will facilitate the removal of foreign bodies from soft tissues. In recent years, oral and maxillofacial surgery cases utilizing both techniques have been reported [7, 10, 15, 16]. The addition of MR technology may help overcome the limitations of navigation systems, such as intraoperative registration and calibration.

## Conclusions

Here, we reported a case demonstrating the successful use of CAD/CAM technology to facilitate the removal of a foreign body embedded in soft tissue.

## Supplementary Information

Supplementary Material 1.

## Data Availability

No datasets were generated or analysed during the current study.

## Abbreviations

CAS: Computer-assisted surgery
CAD/CAM: Computer-aided design/computer-aided manufacturing
CT: Computed tomography
