# Supplementary material for: Using computer-aided design/computer-aided manufacturing technology for foreign body removal from soft tissues: a case report
Source: Maxillofac Plast Reconstr Surg. 2025 Sep 30;47(1):27. doi: 10.1186/s40902-025-00479-4 (PMC12484447; doi:10.1186/s40902-025-00479-4)
Supplement: Supplementary file 1 — Supplementary Material 1. [file 40902_2025_479_MOESM1_ESM.pdf]

CARE case report guidelines

CARE Checklist of information to include when writing a case report

(cc) BY-NC-ND

Topic	Item	Checklist item description	Reported on Line
Title	1	The diagnosis or intervention of primary focus followed by the words "case report"	1page / 1~2 line
Key Words	2	2 to 5 key words that identify diagnoses or interventions in this case report, including "case report"	1page / 23 line
Abstract (no references)	3a	Introduction: What is unique about this case and what does it add to the scientific literature?	1page / 5~8 line
	3b	Main symptoms and/or important clinical findings	N/A
	3c	The main diagnoses, therapeutic interventions, and outcomes	1page / 17~19 line
	3d	Conclusion—What is the main "take-away" lesson(s) from this case?	1page / 22~22 line
Introduction	4	One or two paragraphs summarizing why this case is unique (**may include references**)	2page / 29~34 line
Patient Information	5a	De-identified patient specific information.	2page 37~49 line (5a–5c)
	5b	Primary concerns and symptoms of the patient.	
	5c	Medical, family, and psycho-social history including relevant genetic information	
	5d	Relevant past interventions with outcomes	N/A
Clinical Findings	6	Describe significant physical examination (PE) and important clinical findings.	N/A
Timeline	7	Historical and current information from this episode of care organized as a timeline	2page 37~49 line
Diagnostic Assessment	8a	Diagnostic testing (such as PE, laboratory testing, imaging, surveys).	2page 30~54 line
	8b	Diagnostic challenges (such as access to testing, financial, or cultural)	N/A (8b–10b)
	8c	Diagnosis (including other diagnoses considered)	
	8d	Prognosis (such as staging in oncology) where applicable	
Therapeutic Intervention	9a	Types of therapeutic intervention (such as pharmacologic, surgical, preventive, self-care)	
	9b	Administration of therapeutic intervention (such as dosage, strength, duration)	
	9c	Changes in therapeutic intervention (with rationale)	
Follow-up and Outcomes	10a	Clinician and patient-assessed outcomes (if available)	
	10b	Important follow-up diagnostic and other test results	
	10c	Intervention adherence and tolerability (How was this assessed?)	2~3page / 54~70 page
	10d	Adverse and unanticipated events	N/A
Discussion	11a	A scientific discussion of the strengths AND limitations associated with this case report	4page / 99~105 line
	11b	Discussion of the relevant medical literature **with references**.	6~7page / 146~198 line
	11c	The scientific rationale for any conclusions (including assessment of possible causes)	5page / 127~131 line
	11d	The primary "take-away" lessons of this case report (without references) in a one paragraph conclusion	5page / 127~131 line
Patient Perspective	12	The patient should share their perspective in one to two paragraphs on the treatment(s) they received	N/A
Informed Consent	13	Did the patient give informed consent? Please provide if requested	Yes ☑ No ☐
